# Nef Secretion into Extracellular Vesicles or Exosomes Is Conserved across Human and Simian Immunodeficiency Viruses

**DOI:** 10.1128/mBio.02344-17

**Published:** 2018-02-06

**Authors:** Ryan P. McNamara, Lindsey M. Costantini, T. Alix Myers, Blake Schouest, Nicholas J. Maness, Jack D. Griffith, Blossom A. Damania, Andrew G. MacLean, Dirk P. Dittmer

**Affiliations:** a Department of Microbiology and Immunology, Lineberger Comprehensive Cancer Center, School of Medicine, The University of North Carolina at Chapel Hill, Chapel Hill, North Carolina, USA; b Tulane National Primate Research Center, Tulane University, Covington, Louisiana, USA; Columbia University College of Physicians & Surgeons

**Keywords:** HIV, Nef, SIV, exosomes, extracellular vesicles, microvesicles

## Abstract

Extracellular vesicles (EVs) or exosomes have been implicated in the pathophysiology of infections and cancer. The negative regulatory factor (Nef) encoded by simian immunodeficiency virus (SIV) and human immunodeficiency virus (HIV) plays a critical role in the progression to AIDS and impairs endosomal trafficking. Whether HIV-1 Nef can be loaded into EVs has been the subject of controversy, and nothing is known about the connection between SIV Nef and EVs. We find that both SIV and HIV-1 Nef proteins are present in affinity-purified EVs derived from cultured cells, as well as in EVs from SIV-infected macaques. Nef-positive EVs were functional, i.e., capable of membrane fusion and depositing their content into recipient cells. The EVs were able to transfer Nef into recipient cells. This suggests that Nef readily enters the exosome biogenesis pathway, whereas HIV virions are assembled at the plasma membrane. It suggests a novel mechanism by which lentiviruses can influence uninfected and uninfectable, i.e., CD4-negative, cells.

## IMPORTANCE

Extracellular vesicles (EVs) transfer biologically active materials from one cell to another, either within the adjacent microenvironment or further removed. EVs also package viral RNAs, microRNAs, and proteins, which contributes to the pathophysiology of infection. In this report, we show that both human immunodeficiency virus (HIV) and simian immunodeficiency virus (SIV) incorporate the virus-encoded Nef protein into EVs, including EVs circulating in the blood of SIV-infected macaques and that this presents a novel mechanism of Nef transfer to naive and even otherwise non-infectable cells. Nef is dispensable for viral replication but essential for AIDS progression *in vivo*. Demonstrating that Nef incorporation into EVs is conserved across species implicates EVs as novel mediators of the pathophysiology of HIV. It could help explain the biological effects that HIV has on CD4-negative cells and EVs could become biomarkers of disease progression.

## INTRODUCTION

Extracellular vesicles (EVs) or exosomes have come to be appreciated as a novel and biologically important means of cell-to-cell communications in development, viral and bacterial infections, and cancer (reviewed in reference 1). We use the term EV here to refer to vesicles <100 nm in diameter, containing one or more tetraspanin molecules, and with a characteristic shape observable by electron microscopy (EM). EVs package biologically active materials, such as enzymes, mRNAs, long non-coding RNAs, microRNAs (miRNAs), small-molecule metabolites, etc., and deliver them to recipient cells (2–9). Herpesviruses such as herpes simplex virus 1, Epstein-Barr virus, and Kaposi’s sarcoma-associated herpesvirus incorporate virus-encoded miRNA into EVs (2, 3, 10, 11). Hepatitis A virus incorporates its entire capsid into EVs (5, 12–14), furthering internal spread, while external transmission is mediated by a membraneless virion.

Human immunodeficiency virus type 1 (HIV-1) and HIV-2 entered the human population in the early 20th century from the ancestral simian immunodeficiency virus (SIV) (15, 16). Similar to HIV, SIV establishes chronic infection of the host, ultimately progressing to simian AIDS (sAIDS) in rhesus macaques (*Macaca mulatta*). SIV and HIV belong to the *Retroviridae* family of viruses (genus *Lentivirus*) and infect macrophages and CD4^+^ T cells through defined receptor-coreceptor pairs that are expressed only on these specialized cells; yet, they cause phenotypes dependent on a much larger range of cell types. For instance, SIV and HIV induce a gradual reduction in the level of circulating CD4^+^ lymphocytes over time (15–18). Here, a substantial amount of CD4^+^ T cell death occurs independently of successful viral infection (19, 20). Distinct pathophysiological changes, such as HIV-associated neurocognitive disorders, persist in latently infected individuals or in individuals on successful antiretroviral therapy (ART), implying an indirect mechanism of pathogenesis.

HIV and SIV encode a total of nine genes. A set of early transcripts are fully spliced and encode the accessory proteins Tat, Rev, and Nef. Tat is a potent transcriptional activator that drives the elongation of paused RNA polymerase II, Rev is an RNA-binding protein that facilitates the export of unspliced viral RNAs to the cytoplasm, and Nef is a modulator of the endosomal trafficking network in infected cells (21–26). Nef is a small (~27-kDa) protein that localizes to the cytosol and undergoes several posttranslational modifications, such as myristoylation, that lead to membrane association (18, 27–29). Nef mediates the degradation of the viral receptor CD4 (21, 23, 30–32), and that may be its primary function in productively infected cells.

The importance of Nef becomes visible only within the context of the host. While Tat and Rev are indispensable for viral propagation in cultured T cells, the gene for Nef can be removed entirely or replaced with the gene for green fluorescent protein (GFP) (or other genes) and the resulting recombinant virus retains full replication potential in culture. However, SIV recombinants lacking functional Nef are highly attenuated *in vivo* (21, 33–35) and SIV strains containing point mutations in the *nef* open reading frame rapidly adapt to restore wild-type Nef function upon *in vivo* infection (18, 36, 37). Nef mutations in HIV-infected human patients are overrepresented among natural long-term nonprogressors (38, 39). Nef has been found in the plasma of infected primates and humans (18, 40–45), though not all earlier reports were consistent (35, 43, 44, 46, 47). This suggests that Nef’s role in pathogenesis is not limited to infected cells, but that it could contribute to the more systemic and long-term sequelae of HIV/SIV infection. At that point, a possible interaction between SIV Nef and EVs had not been reported.

We asked if Nef of both HIV and SIV could be detected in secreted EVs. This would establish the conservation of this phenotype and further substantiate the role of the SIV macaque model in HIV research. We were able to demonstrate that (i) the SIV and HIV Nef proteins are consistently present in EVs from transiently transfected cells, (ii) SIV Nef can be detected in systemically circulating EVs of macaques after infection, and (iii) SIV Nef can be transferred to uninfected cells via EVs. Key to our argument for the presence of Nef in EVs was adding a positive affinity purification step that separated EVs from virions, as we had previously validated for EVs and herpesvirus virions (10). These findings support the model in which EVs provide a mechanism for Nef to influence the physiology of uninfected and uninfectable (CD4-negative) cells. The most likely recipients are endothelial cells lining the vascular and lymphatic systems, e.g., of the blood-brain barrier, as these are constantly exposed to EVs that circulate at a concentration as high as 10^11^ particles/ml (48).

## RESULTS

### HIV and SIV Nef proteins are present in EVs released from transfected cells.

**FIG 1:**
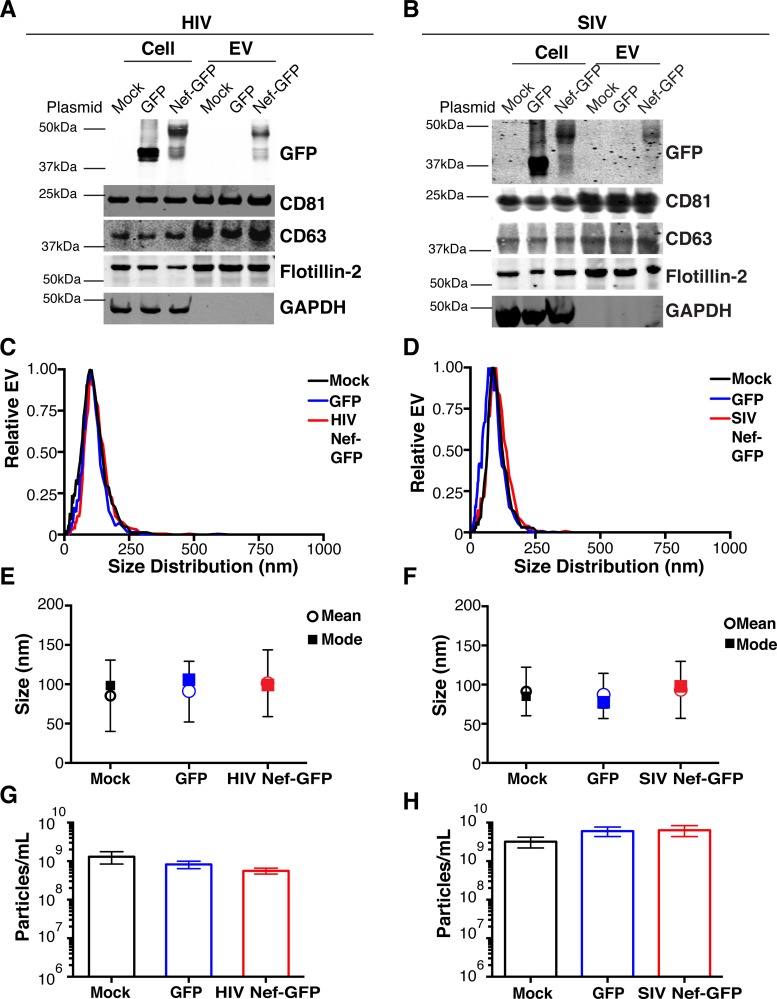
HIV and SIV Nef proteins are present in cell culture-derived EVs. (A) HEK-293 cells were mock transfected or transiently transfected with GFP or HIV *nef*-GFP. Cell pellets were run with equivalent total protein input amounts as determined by GAPDH levels. EV pellets were run with equivalent total numbers of EV particles, as determined by NTA. The EV markers CD81, CD63, and flotillin 2 were used to identify the presence of EVs, and GAPDH was used as an intracellular marker not incorporated into EVs. GFP was used to track both GFP and HIV Nef-GFP. (B) HEK-293 cells were mock transfected or transiently transfected with GFP or SIV *nef*-GFP. Cell pellets were run with equivalent total protein input amounts, as determined by GAPDH levels. EV pellets were run with equivalent total numbers of EV particles, as determined by NTA. The EV markers CD81, CD63, and flotillin 2 were used to identify the presence of EVs, and GAPDH was used as an intracellular marker not incorporated into EVs. GFP was used to track both GFP and SIV Nef-GFP. (C) Size distribution analysis of EVs isolated from cells transfected as described for panel A by NTA with the Particle Metrix ZetaView. Videos of EV populations were taken to determine size distributions (11 measurements per group with a total of four biological replicates). The peak size was arbitrarily set to 1 for each group. (D) Size distribution analysis of EVs isolated from cells transfected as described for panel B by using the same methods as for panel C. (E) Mean and mode sizes of EVs from cells transfected as described for panel A. *n* = 4 per group. (F) Mean and mode sizes of EVs from cells transfected as described for panel B. *n* = 4 per group. (G) Total EV concentration (particles per milliliter) of cell culture supernatant from cells transfected as described for panel A. *n* = 4 per group. (H) Total EV concentration (particles per milliliter) of cell culture supernatant from cells transfected as described for panel B. *n* = 4 per group. See also Fig. S1.

To test the hypothesis that Nef could be incorporated into EVs independently of other viral components, we transiently expressed the HIV and SIV Nef proteins in human embryonic kidney (HEK-293) cells. We used Nef tagged with GFP to monitor transfection efficiency. As an epitope tag control, we transfected wild-type GFP alone. HIV Nef-GFP, as well as SIV Nef-GFP, but not GFP alone, was detectable in the EV fraction (Fig. 1A and B). See Materials and Methods for the details of the EV purification protocol used, which is similar to that described in reference 49. We used the tetraspanin markers CD81 and CD63, as well as EV-associated flotillin 2 (50, 51), as markers for EV purity and loading controls and cytosolic glyceraldehyde-3-phosphate dehydrogenase (GAPDH) as a control for contamination with cytosolic proteins. We verified the biophysical properties of EV fractions by nanoparticle tracking analysis (NTA). Through zeta potential and Brownian motion, we were able to plot the relative size distribution of the isolated EVs. Regardless of the cell origin, the EV size distribution profiles were highly similar and showed a canonical curve shape (2, 3, 10, 49, 52, 53) (Fig. 1C and D; see Movies S1 to S6 in the supplemental material). Mean and mode sizes were consistent across all experiments (Fig. 1E and F). Total EV release did not differ among cells expressing HIV Nef-GFP (Fig. 1G) or SIV Nef-GFP (Fig. 1H) compared to GFP- or mock-transfected cells. This phenotype was repeatable with cells transfected with hemagglutinin (HA) epitope-tagged HIV Nef, demonstrating that Nef’s presence in EVs was not due to being tagged with a cytoplasmic protein such as GFP (Fig. S1). These experiments demonstrate that the HIV and SIV Nef proteins are loaded into EVs in the absence of other virus components.

10.1128/mBio.02344-17.3 MOVIE S1  Nanoparticle tracking video of EVs isolated from mock-transfected HEK-293 cells. Total EVs from mock-transfected HEK-293 cells were analyzed by the ZetaView from Particle Metrix. Particle concentration was determined by the number of tracked particles, and size distribution was determined by Brownian motion (85). Download MOVIE S1, MOV file, 8.4 MB. Copyright © 2018 McNamara et al. 2018 McNamara et al. This content is distributed under the terms of the Creative Commons Attribution 4.0 International license.

10.1128/mBio.02344-17.4 MOVIE S2  Nanoparticle tracking video of EVs isolated from GFP-transfected HEK-293 cells. Total EVs from GFP-transfected HEK-293 cells were analyzed by the ZetaView from Particle Metrix. Particle concentration was determined by the number of tracked particles, and size distribution was determined by Brownian motion. Download MOVIE S2, MOV file, 8.4 MB. Copyright © 2018 McNamara et al. 2018 McNamara et al. This content is distributed under the terms of the Creative Commons Attribution 4.0 International license.

10.1128/mBio.02344-17.5 MOVIE S3  Nanoparticle tracking video of EVs isolated from HIV *nef*-GFP-transfected HEK-293 cells. Total EVs from HIV *nef*-GFP-transfected HEK-293 cells were analyzed by the ZetaView from Particle Metrix. Particle concentration was determined by the number of tracked particles, and size distribution was determined by Brownian motion. Download MOVIE S3, MOV file, 8.3 MB. Copyright © 2018 McNamara et al. 2018 McNamara et al. This content is distributed under the terms of the Creative Commons Attribution 4.0 International license.

10.1128/mBio.02344-17.6 MOVIE S4  Nanoparticle tracking video of EVs isolated from mock-transfected HEK-293 cells. Total EVs from mock-transfected HEK-293 cells were analyzed by the ZetaView from Particle Metrix. Particle concentration was determined by the number of tracked particles, and size distribution was determined by Brownian motion. Download MOVIE S4, MOV file, 8.3 MB. Copyright © 2018 McNamara et al. 2018 McNamara et al. This content is distributed under the terms of the Creative Commons Attribution 4.0 International license.

10.1128/mBio.02344-17.7 MOVIE S5  Nanoparticle tracking video of EVs isolated from GFP-transfected HEK-293 cells. Total EVs from GFP-transfected HEK-293 cells were analyzed by the ZetaView from Particle Metrix. Particle concentration was determined by the number of tracked particles, and size distribution was determined by Brownian motion. Download MOVIE S5, MOV file, 8.4 MB. Copyright © 2018 McNamara et al. 2018 McNamara et al. This content is distributed under the terms of the Creative Commons Attribution 4.0 International license.

10.1128/mBio.02344-17.8 MOVIE S6  Nanoparticle tracking video of EVs isolated from SIV *nef*-GFP-transfected HEK-293 cells. Total EVs from SIV *nef*-GFP-transfected HEK-293 cells were analyzed by the ZetaView from Particle Metrix. Particle concentration was determined by the number of tracked particles, and size distribution was determined by Brownian motion. Download MOVIE S6, MOV file, 8.1 MB. Copyright © 2018 McNamara et al. 2018 McNamara et al. This content is distributed under the terms of the Creative Commons Attribution 4.0 International license.

10.1128/mBio.02344-17.1 FIG S1  HIV Nef incorporation into EVs is epitope independent. (A) HEK-293 cells were mock transfected (empty vector) or transiently transfected with HIV Nef-HA. Cell and EV pellets were subjected to Western blot analysis for HA and the EV marker CD81. (B) Size distribution analysis of EVs isolated from cells transfected as described for panel A by NTA with the Particle Metrix ZetaView. Videos of EV populations were taken to determine size distributions (11 measurements per group with a total of four biological replicates). The peak size was arbitrarily set to 1 for each group. (C) Mean and mode sizes of EVs from cells transfected as described for panel A. *n* = 4 per group. (D) Total EV concentration (particles per milliliter) of cell culture supernatant from cells transfected as described for panel A. *n* = 4 per group. Download FIG S1, TIF file, 0.8 MB. Copyright © 2018 McNamara et al. 2018 McNamara et al. This content is distributed under the terms of the Creative Commons Attribution 4.0 International license.

### Nef and the EV marker CD81 colocalize.

**FIG 2:**
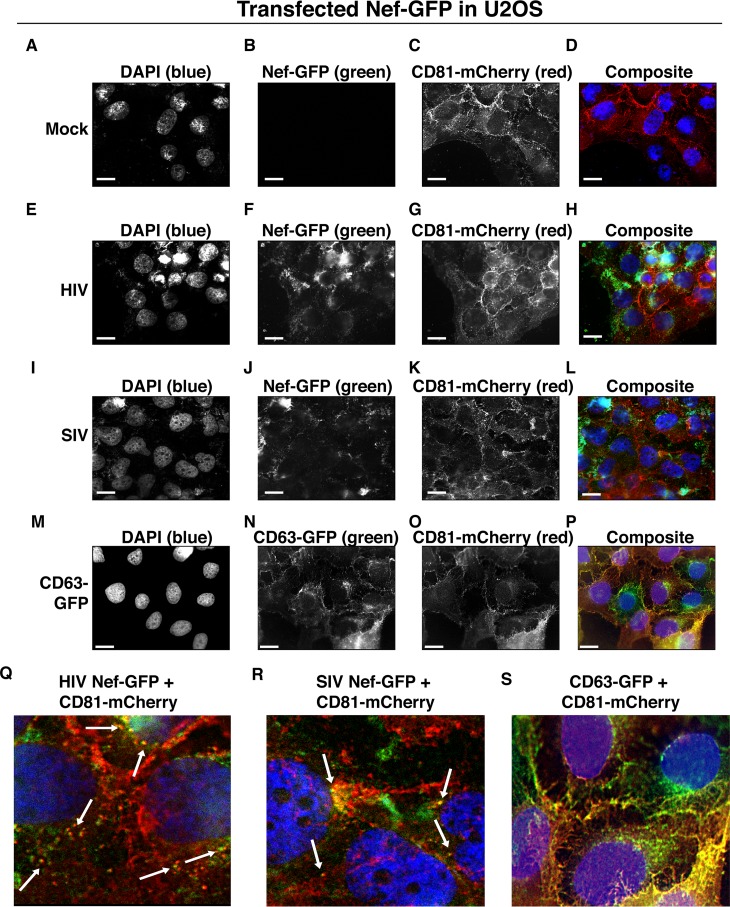
HIV and SIV Nef proteins colocalize in the cytoplasm with the EV marker CD81 in cells. (A to D) U2OS cells were selected to stably express the EV marker CD81-mCherry and then mock transfected (empty vector). Single-plane images from deconvoluted z-stacks were used to visualize DAPI (A), GFP (B), CD81-mCherry (C), and a composite (D). Size bars = 200 μm. (E to H) Same as panels A to D but for cells transfected with HIV *nef*-GFP. Single-plane images from deconvoluted z-stacks were used to visualize DAPI (E), GFP (F), CD81-mCherry (G), and a composite (H). (I to L) Same as panels A to D but for cells transfected with SIV *nef*-GFP. Single-plane images from deconvoluted z-stacks were used to visualize DAPI (I), GFP (J), CD81-mCherry (K), and a composite (L). (M to P) U2OS cells constitutively expressing CD63-GFP and CD81-mCherry were used as a positive control. Single-plane images from deconvoluted z-stacks were used to visualize DAPI (M), CD63-GFP (N), CD81-mCherry (O), and a composite (P). (Q to S) Selected areas from U2OS CD81-mCherry-expressing cells coexpressing HIV Nef-GFP (Q), SIV Nef-GFP (R), and CD63-GFP enlarged to show areas of colocalization events (S).

To test the hypothesis that Nef colocalizes with EV components during EV maturation, we employed fluorescence microscopy. Exosomes are derived from the inward budding of endosomes into the multivesicular body (MVB), trafficked from there to the plasma membrane, and released (52, 54). Therefore, EV components such as CD63, CD81, CD9, and flotillin 2 can be used to mark maturing late endosomes/MVBs inside the cell. We generated U2OS cells, which constitutively express CD81-mCherry (Fig. 2A to D). We then used these cells to transiently express HIV Nef-GFP (Fig. 2E to H) or SIV Nef-GFP (Fig. 2I to L). As a control, we analyzed patterns of colocalization of CD81-mCherry and CD63-GFP (Fig. 2M to P). Diffuse patterns of localization, as well as several regions of punctate colocalization events, could be identified involving CD81-mCherry and both HIV Nef (Fig. 2Q) and SIV Nef (Fig. 2R) and in our CD63-GFP positive control (Fig. 2S). This is consistent with the notion that Nef enters the EV maturation pathway and eventually resides inside EVs.

### Nef is transferred to recipient cells by EVs.

**FIG 3:**
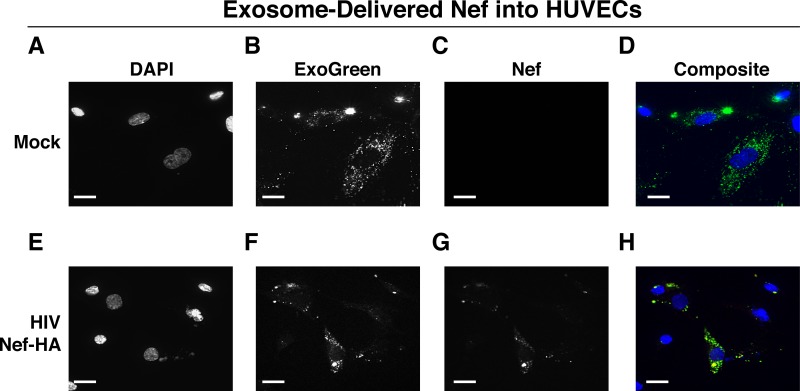
HIV Nef can be transferred to endothelial cells by EVs. hTERT-HUVECs were treated with ExoGreen-labeled EVs. ExoGreen is a pan-EV label used to track the uptake of EVs by cells. Scale bars = 100 μm. (A to D) Cells were given EVs taken from transiently mock-transfected HEK-293 cells. (E to H) Cells were given EVs from transiently Nef-HA-transfected HEK-293 cells. Single-plane images from deconvoluted z-stacks were used to visualize DAPI (E), ExoGreen (F), Nef-HA (G), and a composite (H). Scale bars = 200 μm.

To test the hypothesis that EV-associated Nef was released and transferred to target cells, we conducted fusion assays. EVs were purified from HEK-293 cells transiently expressing HIV Nef-HA and added to human telomerase reverse transcriptase-human umbilical vein endothelial cells (hTERT-HUVECs). HUVECs are a physiologically relevant target because endothelial cells lining the vasculature are constantly exposed to all EVs released into the circulation. Levels of EVs circulating in the blood have been estimated at 10^10^ to 10^12^/ml (55). Upon the addition of ExoGreen-labeled EVs, green punctate structures representing EV fusion are readily apparent (Fig. 3A to D). Only in cells treated with EVs taken from HIV Nef-HA-transfected cells, not in cells treated with EVs taken from mock-transfected cells, was an HA signal also apparent (Fig. 3E to H). The Nef-HA signal (red) was observed only in the presence of colocalizing ExoGreen (pan-EV stain); while the ExoGreen signal was also observed in areas without Nef. This was expected, as not all EVs also contain Nef, even when overexpressed, and demonstrated that Nef can be transferred into recipient cells via EVs.

### Nef is detectable in EVs purified from SIV-infected macaques.

**FIG 4:**
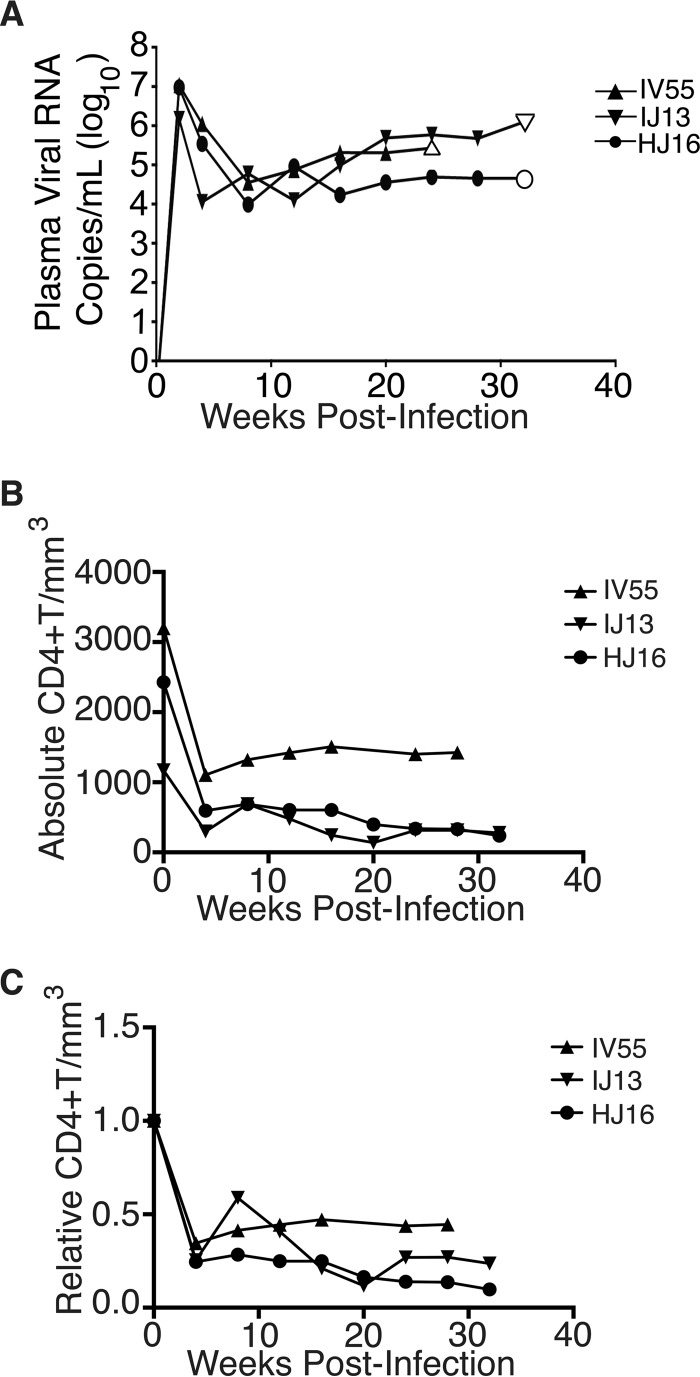
Time course of SIV infection in macaques. (A) Indian origin rhesus macaques maintained at the TNPRC were inoculated with SIVmac239, blood was harvested, and circulating viral RNA was quantified throughout the study. Open symbols represent the times at which animals were sacrificed. (B) Absolute CD4^+^ T cell counts of rhesus macaques throughout SIV infection. Blood was taken from macaques pre- and postinfection with SIV, and CD4^+^ T counts were determined by flow cytometry. (C) Relative CD4^+^ T cell counts of rhesus macaques throughout SIV infection. Blood was taken from macaques pre- and postinfection with SIV, and CD4^+^ T counts were standardized to day zero for each animal.

The biochemical experiments established that loading into EVs and transfer were conserved in the SIV and HIV Nef proteins. To test the hypothesis, that SIV Nef was systemically circulating during natural SIV infection, three rhesus macaques (IV55, IJ13, and HJ16) were infected with SIVmac239. The animals were monitored for viral load and disease progression for 32 weeks. Plasma was obtained from the animals pre- and postinfection, and viral RNA was quantified by quantitative reverse transcription (qRT)-PCR (Fig. 4A). Chronic-phase set point (>10 weeks postinfection) SIV titers ranged from 10^5^ to 10^7^ copies/ml, which is the expected range for rhesus macaques infected with SIVmac239 (56). Although the initial levels of CD4^+^ T cells differed among the animals, they all exhibited the typical steep decline in lymphocytes shortly after infection and their CD4 levels remained low throughout the time course (Fig. 4B). Adjustment for relative CD4^+^ T lymphocyte levels prior to infection for each individual animal yielded consistent results for the duration of infection (Fig. 4C).

**FIG 5:**
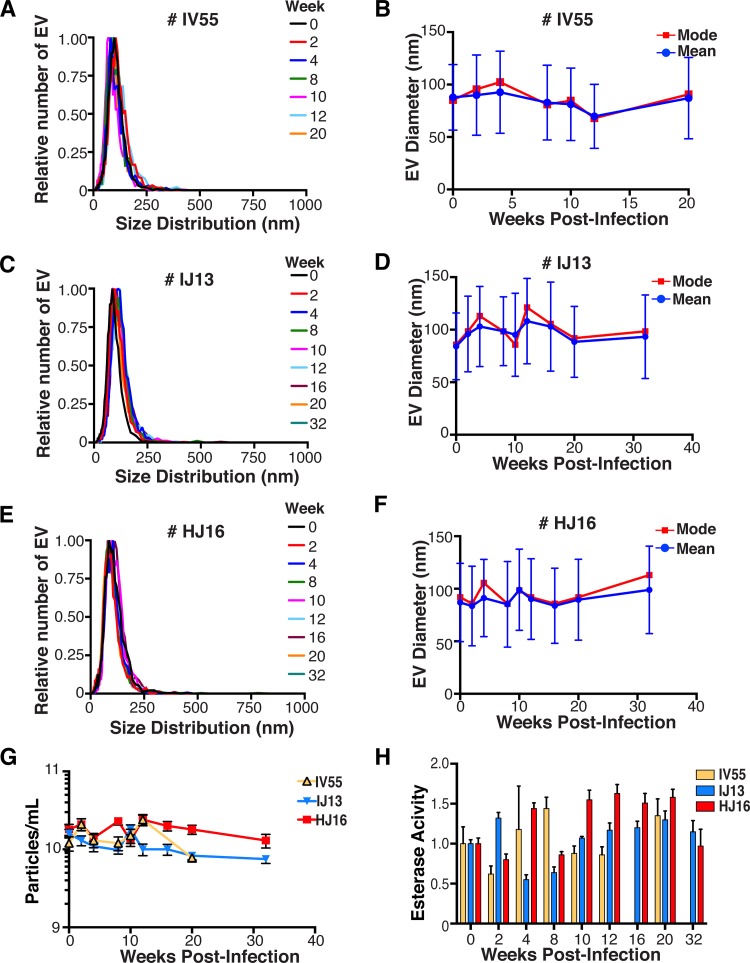
Quantitative analysis of the EV fraction from macaque plasma. EVs were isolated from 3 ml of plasma from macaques IV55 (A), IJ13 (C), and HJ16 (E) and analyzed with the ZetaView from Particle Metrix. Relative size distribution was analyzed by Brownian motion (85) and plotted for EVs taken from the animals preinfection (week 0) and at various time points postinfection with SIV (weeks 2 to 32). Mode (red) and mean (blue) sizes of EVs isolated from the plasma of macaques IV55 (B), IJ13 (D), and HJ16 (F) were determined from the size distribution analysis. The mode and mean sizes of the EVs are shown preinfection (week 0) and at various time points postinfection with SIV (weeks 2 to 32). (G) Total EV concentrations (particles per milliliter) in the plasma of the three animals preinfection (week 0) and at various time points postinfection with SIV (weeks 2 to 32) (11 measurements per group). (H) Esterase activity of EVs isolated from the plasma of the three animals preinfection (week 0) and at various time points postinfection with SIV (weeks 2 to 32). All values are standardized to week 0 for each animal (*n* = 4 for each time point for each animal).

EVs were isolated from plasma. Size distribution (Fig. 5A) and mean and mode EV diameters (Fig. 5B) were similar to those previously observed for human EV preparations (49, 57), as well as our *in vitro* isolation of EVs (compare Fig. 1 and 5). No significant differences in size between pre- and postinfection samples were observed. Similar results were obtained for animals IJ13 (Fig. 5C and D) and HJ16 (Fig. 5E and F). We measured a concentration of ~10^10^ EVs/ml (compared to ~10^6^ SIV copies/ml). No consistent trend in the plasma EV concentration, as measured by NTA, emerged in the three animals (Fig. 5G). As an alternative approach to the quantitation of EVs, we monitored enzymatic activity from the EV-packaged esterases (58). No apparent trend was observed in the three animals throughout the course of infection (Fig. 5H). This demonstrates that EVs are present in plasma and that intact, enzymatically active EVs can be purified from plasma samples.

**FIG 6:**
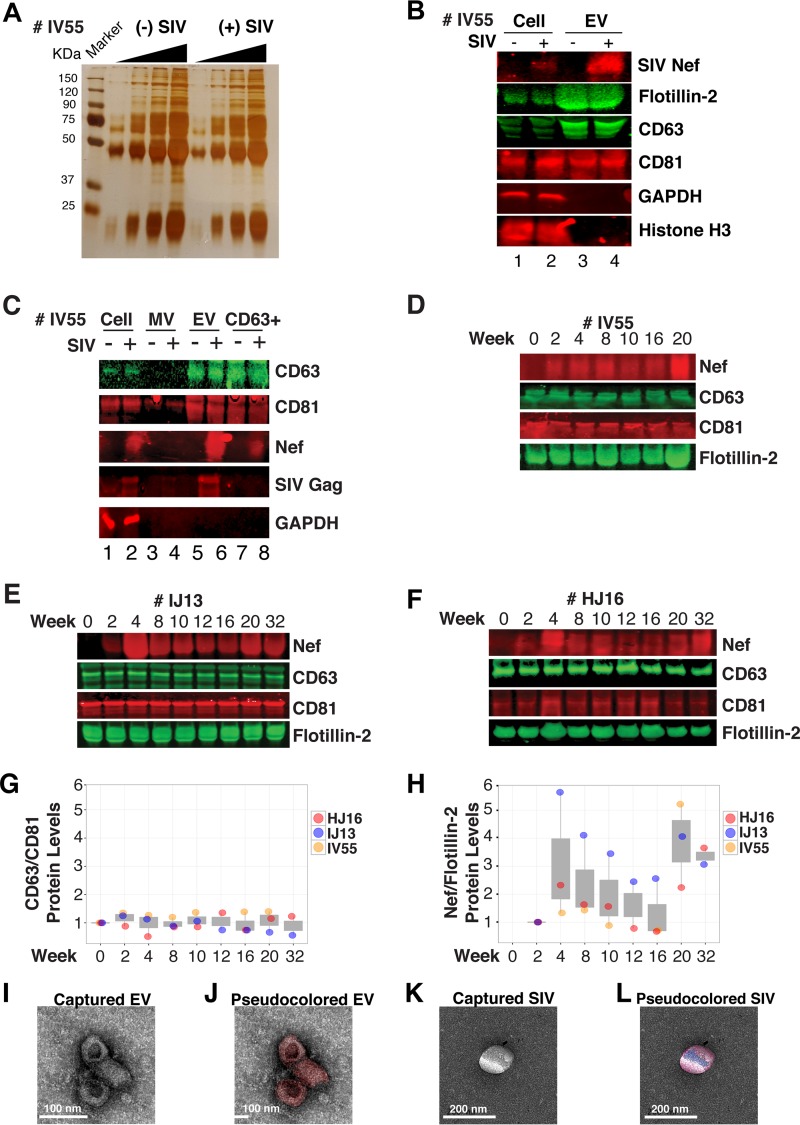
Nef is a constituent of CD63^+^ EVs *in vivo*. (A) Silver stain analysis of total EVs isolated from macaque IV55 pre- and postinfection with SIV. EVs were diluted to equivalent concentrations (1 × 10^9^, 2 × 10^9^, 4 × 10^9^, and 8 × 10^9^ particles/ml), and contents were run for silver stain analysis. (B) Nef is detected in the EV fraction from SIV-infected macaques. Cell pellets and EV fractions from animal IV55 pre- and postinfection with SIV were assayed for the presence of Nef. Cell pellets were run with equivalent total protein input amounts, as determined by GAPDH and histone H3 levels. EV pellets were run with equivalent total numbers of EV particles, as determined by NTA. The EV markers flotillin 2, CD63, and CD81 were used to identify the presence of EVs. The SIVmac239 Nef antibody was used to track Nef. (C) Nef is present in CD63^+^ affinity-purified EVs. Cell pellet, MV, total EV, and CD63/CD81 affinity-purified fractions were assayed for the presence of Nef and other protein components. (D) Nef levels in CD63^+^ EVs throughout the course of infection of macaque IV55. EVs were CD63^+^ affinity purified at various time points pre- and postinfection with SIV. (E) Same as panel D but for IJ13. (F) Same as panel D but for HJ16. (G) Quantitation of the CD63/CD81 levels of the three animals throughout infection. (H) Quantification of the Nef/flotillin 2 levels of the three animals throughout infection. (I) Representative electron micrograph of CD63^+^ EVs from animal IV55. Scale bar = 100 nm. (J) CD63^+^ EVs imaged in panel I pseudocolored red to improve contrast. Scale bar = 100 nm. (K) Representative electron micrograph of SIV particle from animal IV55. Scale bar = 200 nm. (L) SIV particle imaged in panel J pseudocolored blue and red for contrast. Scale bar = 200 nm. See also Fig. S2.

Next, we analyzed total EV protein content by silver stain analysis, loading increasing concentrations from each sample (maximum loading, 10^8^ particles). No major protein band differences between the postinfection samples and the preinfected controls were present (Fig. 6A). SIV Nef was detected in the cell and EV pellet from SIV-infected macaque IV55 but not in the EV pellet from the same animal prior to infection (Fig. 6B, compare lanes 1 and 2 and lanes 3 and 4). Of note, the antibody raised against native SIV Nef exhibited a much lower sensitivity than the commercial antibodies that were raised against the dedicated biochemical tag GFP (Fig. S2). For that reason, we had to greatly extend the exposure times for Nef detection and would only be able to detect Nef if the protein was present at levels above those found in EVs purified from Nef-transfected cells in culture. GAPDH, which is a cytosolic protein, and histone H3, which is a nuclear protein, were detected in the cell pellet but not in purified EVs (Fig. 6B, compare lanes 1 and 2 with lanes 3 and 4). The canonical EV markers CD63 and CD81 and the endosome-derived membrane protein flotillin 2 were present in the EVs (Fig. 6B, lanes 3 and 4). This experiment demonstrates that SIV Nef is present within EVs from SIV-infected macaques.

10.1128/mBio.02344-17.2 FIG S2  Detection of Nef with anti-Nef antibody is far less sensitive than with anti-GFP antibody. Lysates of transiently SIV Nef-transfected HEK-293 cells were electrophoresed on a gel and probed with a SIV Nef or GFP antibody. Nef blot assays are from the same exposure. β-Actin was used as a standardizing control. Download FIG S2, TIF file, 0.3 MB. Copyright © 2018 McNamara et al. 2018 McNamara et al. This content is distributed under the terms of the Creative Commons Attribution 4.0 International license.

Virions may cosediment with EVs upon differential centrifugation (58, 59). In the case of HIV, it has been speculated that the HIV Nef signal observed in EV preparations was due to contaminating HIV particles, particularly if EVs were purified from high-HIV-titer cell culture supernatant (21, 45, 60). Similar concerns had been raised upon the demonstration that herpesviral miRNA is present in EVs purified from cell culture supernatant, plasma, or effusion fluid (10). To address this concern, EVs were affinity purified with magnetic beads containing antibodies directed toward EV membrane proteins (see Materials and Methods). By this approach, virions flow through the column while EVs are captured and eluted after extensive washing. After this additional purification step was added, Nef, but not the SIV Gag protein, was detected in EVs (Fig. 6C, lanes 7 and 8). Hence, positive affinity purification was able to separate EVs from SIV virions. This result was consistent, as affinity-purified EVs obtained from the three animals (Fig. 6D to F) at multiple time points of infection all contained Nef (Fig. 6G and H). Nef was not detectable in the microvesicle (MV) fraction (Fig. 6C, lanes 3 and 4), consistent with our initial observation that Nef entered the EV biogenesis pathway rather than being released during cell death. To verify the purity of the EVs, we used transmission EM (TEM). We could recognize particles of typical EV morphology in the EV fraction (Fig. 6I and J) and SIV particles of prototypical morphology in the flowthrough fraction (Fig. 6K and L). Particles observed by TEM were consistent with the established sizes of exosomes (30 to 100 nm) or SIV virions (~120 to 150 nm) (61, 62). These data support the notion that Nef is present in EVs during natural SIV infection.

### EVs purified from macaque plasma retain biological activity.

**FIG 7:**
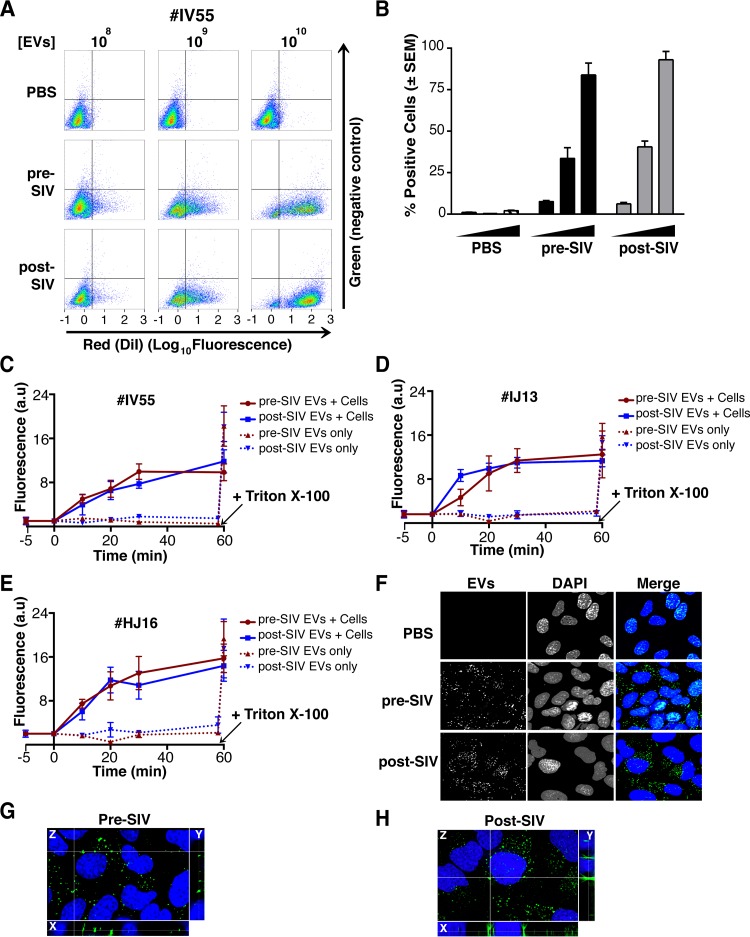
EVs taken from macaque plasma are competent for adsorption and deposit packaged materials into recipient cells. (A) EVs were labeled with the membrane dye DiI and added to recipient cells in a dose-dependent manner. Flow cytometry analysis of U2OS (recipient) cells shows that the fluorophore was transferred by EVs taken from animal IV55 pre- and postinfection with SIV but not by PBS (*T* = 8 h). (B) Plot of the percentages of positive cells gated for panel A. (C) EVs taken from IV55 are capable of membrane fusion. EVs taken pre- and postinfection with SIV were labeled with the self-quenching dye R18 and added to cells or empty wells. Fluorescence was monitored over time. As a control, Triton X-100 was added to the EVs in empty wells to disperse the fluorophore at the plateau phase. (D) Same as panel C but for EVs from IJ13. (E) Same as panel C but for EVs from HJ16. (F) EVs taken from animal IV55 pre- and postinfection with SIV were labeled with the esterase reporter ExoGlow Green, added to cells, and monitored for adsorption. Single-plane images were deconvoluted from three-dimensional Z-stacks. ExoGlow Green was added to PBS as a negative control. (G) Enlarged three-dimensional view of cells treated with EVs taken from animal IV55 preinfection with SIV. (H) Enlarged three-dimensional view of cells treated with EVs taken from animal IV55 postinfection with SIV. a.u., arbitrary units.

To demonstrate that the purified EVs retain biological activity, we assayed their ability to fuse with recipient cells by multiple independent means. (i) EVs were fluorescently tagged with the membrane dye 1,1′-didodecyl-3,3,3′,3′-tetramethylindocarbocyanine perchlorate (DiI; see Materials and Methods). By flow cytometry, fluorophore transfer from purified EVs to target cells was observed in a dose-dependent manner (Fig. 7A and B). This was not observed when using cells treated with phosphate-buffered saline (PBS) plus DiI (without EVs), demonstrating that the marker was incorporated into EV membranes and carried through during the purification steps. (ii) EVs were labeled with the self-quenching dye R18 (octadecyl rhodamine chloride), which fluoresces only when the donor (labeled) membrane fuses with a recipient membrane (63). Fluorophore transfer from purified EVs to target cells was established by a temporal increase in fluorescence (Fig. 7C, solid lines). As a positive control, Triton X-100 was added at the end of the experiment, which resulted in the expected fluorescence increase in all wells containing EVs (Fig. 7C, dashed lines; see Materials and Methods). No differences were observed in the fusion rates of EVs pre- and postinfection of the three animals infected with SIV (Fig. 7C to E). (iii) To test if EV contents were taken up by cells, EVs were incubated with a membrane-permeating ExoGreen esterase reporter, which fluoresces green and covalently links to proteins inside EVs. Addition of labeled EVs, but not our PBS control, to cells showed intracellular uptake of the reporter (Fig. 7F to H). The punctate formation of the proteins delivered by EVs likely represents larger EV aggregates and not end point compartmentalized proteins *per se*. This was consistent with delivered EVs taken from a tissue culture setting. This assay demonstrated that this EV purified from plasma contained the EV-defining, enzymatically active esterases and remained competent for membrane fusion and transfer of proteins into recipient cells.

### EVs purified from naturally infected macaques transfer SIV Nef to recipient cells.

**FIG 8:**
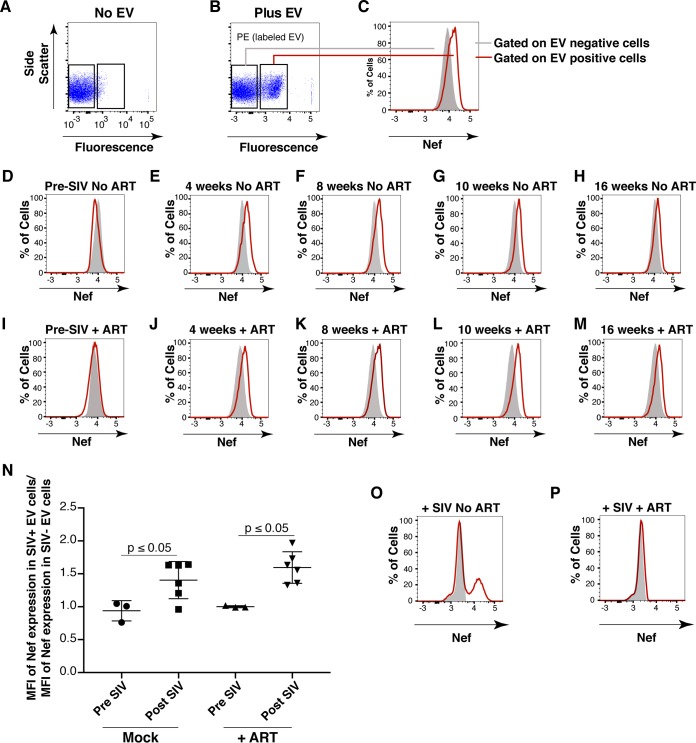
Detection of Nef in EV-transduced cells. (A) Primary CD4^+^ T cells were analyzed for intrinsic fluorescence (PE gate) and gated. (B) Primary CD4^+^ T cells treated with labeled EVs were gated into EV-positive and EV-negative populations on the basis of fluorescence. (C) Representative scheme for detection of Nef (FITC channel) by using the gates set up for panel B. Solid gray fill represents EV-negative cells, and a red line represents EV-positive cells. (D) EVs isolated from macaque IV55 preinfection with SIV were labeled and added to purified primary CD4^+^ T cells. Cells were gated as for panels B and C, and the FITC shift was absent (no Nef). EVs isolated from macaque IV55 at weeks 4 (E), 8 (F), 10 (G), and 16 (H) postinfection with SIV were added to CD4^+^ T cells. Cells were gated as for panels B and C, and the FITC shift (Nef) in EV-positive cells was observed. (I) EVs isolated from macaque IV55 preinfection with SIV were labeled and added to CD4^+^ T cells in the presence of ART drugs. Cells were gated as for panels B and C, and the FITC shift was absent (no Nef). EVs isolated from macaque IV55 at weeks 4 (J), 8 (K), 10 (L), and 16 (M) postinfection with SIV were added to CD4^+^ T cells in the presence of ART drugs. Cells were gated as for panels B and C, and the FITC shift (Nef) in EV-positive cells was observed. (N) Plot comparing the MFI of the Nef signals in EV^+^ and EV^−^ cells compiling all time points after SIV infection in independent experiments. Each symbol represents the MFI of Nef in EV^+^ cells divided by the MFI of Nef in EV^−^ cells in the same well. The ratios cluster around 1 at preinfection time points, verifying that Nef is absent from EVs isolated from animal IV55 before SIV infection. (O) Primary CD4^+^ T cells were mock treated (solid gray) or infected with SIVmac239 (red line), and an FITC shift (Nef) was detected only in infected cells. (P) Same as panel O but cells were treated with ART drugs to block infection.

To test the hypothesis that SIV Nef from infected animals in EVs can be transferred into recipient cells, we used a flow cytometry-based assay optimized for intracellular Nef staining (64). This was a rather difficult experiment that approached the limits of sensitivity, as Nef was neither overexpressed nor tagged. Treatment of purified primary CD4^+^ T cells from SIV-naive macaques with fluorescently labeled EVs derived from uninfected macaques demonstrated successful transfer of the fluorophore [phycoerythrin (PE) channel], signifying that EVs adsorbed to the cells at high rates (Fig. 8A and B). We then gated for cells that showed uptake of EVs versus those that did not and used the fluorescein isothiocyanate (FITC) channel to detect intracellular Nef (Fig. 8C). As expected, no shift in mean FITC fluorescence was detected in cells treated with EVs taken from macaques before SIV infection (Fig. 8D). In cells treated with EVs isolated from macaques postinfection with SIV, a shift in the FITC (Nef) channel was observed at multiple time points throughout infection (Fig. 8E to H). The addition of the ART drugs zidovudine (AZT; reverse transcriptase inhibitor) and nelfinavir (NFV; protease inhibitor) did not antagonize the FITC (Nef) shift observed postinfection with SIV in EV-positive cells relative to EV-negative cells (Fig. 8I to M), demonstrating that the Nef signal did not originate from new infections. Mean fluorescence intensities were clustered into pre- or postinfection in the absence (Mock) or presence of ART drugs, and statistical significance (*P* < 0.05) was calculated with pairwise *t* tests (Fig. 8N). These findings were in contrast to those obtained with cells directly infected with SIVmac239 (Fig. 8O), in which Nef detection was abolished when cells were treated with the ART drug cocktail (Fig. 8P). Taken together, the results show that ART abolished the detection of Nef in the context of SIV infection but had no impact on Nef detection in the context of EV-mediated transfer. This experiment demonstrates that SIV Nef purified from naturally infected animals can be transferred to recipient cells by an EV.

### PBMCs infected with SIV incorporate Nef into EVs.

**FIG 9:**
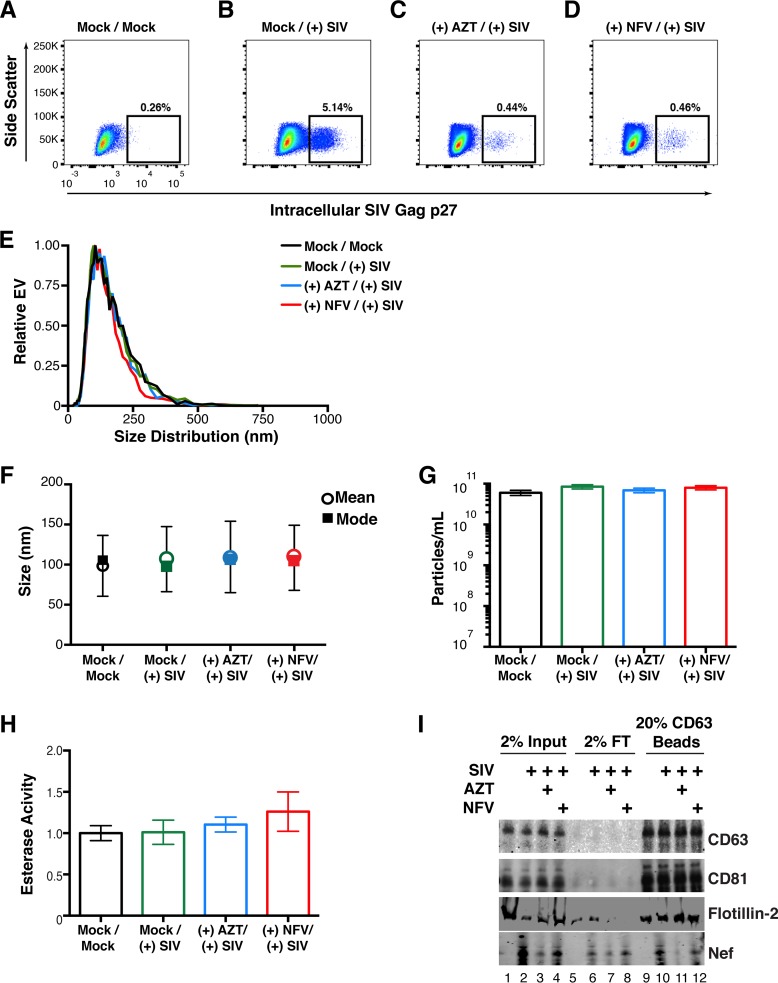
Detection of Nef in EVs from SIV-infected primary cells. (A) Primary PBMCs were isolated from rhesus macaques, mock treated, and stained for intracellular SIV Gag (p27) after 5 days. (B) Primary PBMCs were isolated from rhesus macaques, infected with SIVmac239 at an MOI of 10, and stained for intracellular SIV Gag 5 days postinfection. (C) Primary PBMCs were isolated from rhesus macaques and treated with the reverse transcriptase inhibitor AZT (100 nM). Twenty-four hours later, the cells were infected with SIVmac239 at an MOI of 10 and stained for intracellular SIV Gag 5 days postinfection. (D) Primary PBMCs were isolated from rhesus macaques and treated with the viral protease inhibitor NFV (100 nM). Twenty-four hours later, the cells were infected with SIVmac239 at an MOI of 10 and stained for intracellular SIV Gag 5 days postinfection. (E) Size distribution analysis of EVs isolated from the supernatant of simian macaque primary PBMCs infected with SIVmac239 (see Materials and Methods). Videos of EV populations were taken to determine size distributions (11 measurements per group with a total of three biological replicates). The peak size was arbitrarily set to 1 for each group. (F) Mean and mode sizes of EVs from the PBMCs treated for panel A. *n* = 3 per group. (G) Total EV concentration (particles per milliliter) in supernatant of PBMCs treated for panel A. *n* = 3 per group. (H) Esterase activity of EVs isolated from the PBMCs treated for panel A. All values are standardized to the Mock/Mock group. *n* = 3 per group. (I) SIV Nef is present in CD63^+^ affinity-purified EVs taken from infected PBMCs. Total EVs from panel A were added to CD63 antibody-coated beads and assayed for the presence of Nef. Input, total EV population; flowthrough (FT), unbound fraction; CD63 Beads, contents bound to CD63 beads.

To assay if we could recapitulate our *in vivo* infections of macaques in an *in vitro* model, we isolated peripheral blood mononuclear cells (PBMCs) from four SIV-naive macaques (see Materials and Methods). The cells were activated with concanavalin A (ConA) and interleukin-2 (IL-2) for 48 h and then mock treated or treated with the reverse transcriptase inhibitor AZT or the viral protease inhibitor NFV. The cells were then infected with SIVmac239 at a multiplicity of infection (MOI) of 10. Detection of intracellular Gag was done by flow cytometry and showed successful infection of the cells. Importantly, the percentage of cells that stained positive for SIV Gag p27 was much lower after treatment with AZT and NFV (Fig. 9A to D). This was expected, as virus infection is inhibited in cells treated with AZT and maturation of the virus particle is inhibited in cells treated with NFV. However, full inhibition of p27 detection was not accomplished by these two methods (see below).

The supernatant of the cells was harvested, and total EVs were isolated. The size distribution profiles of the treatment groups were similar (Fig. 9E), and no changes in mean or mode sizes were identified (Fig. 9F). The concentrations of EVs (Fig. 9G) and their enzymatic activity were remarkably similar as well (Fig. 9H). We next asked if Nef could be captured by CD63 antibody-coated beads, similar to the approach used in our *in vivo* macaque infections. Tetraspanins such as CD63 and CD81 were readily detected in the total EV fraction (input) of all of our treatment groups, as was the EV marker flotillin 2 (lanes 1 to 4). Nef was present only in SIV-infected samples, and its presence was greatly reduced in cells treated with AZT (lane 3) and to a lesser extent in cells treated with NFV (lane 4). Nef was present in the flowthrough fraction (lanes 6 to 8), indicating that not all Nef is present in CD63^+^ EVs, consistent with previous reports of the protein being present in viral particles (18, 45). We were able to detect Nef in the CD63^+^ EV fraction of SIV-infected PBMCs (lane 10) and at lower levels in cells treated with AZT (lane 11) and NFV (lane 12). The detection of Nef in AZT-treated samples was likely due to the inability of the drug to completely block infection; detection of Nef in NFV-treated cells was expected, as the drug targets the maturation of the viral particle, which occurs long after Nef production.

## DISCUSSION

The role of EVs, particularly exosomes (EVs ≤100 nm in diameter with a defined maturation pathway), in virus infection has received considerable attention in recent years. Exosomes are a class of EVs that originate from the inward budding of endosomes into the MVB and subsequent release into the supernatant (reviewed in references 1 and 52). The mechanism of incorporation of Nef into EVs is currently unknown. We suspect that this incorporation occurs within maturing late endosomes and/or the MVB on the basis of our analysis of Nef colocalization with CD81, although a higher-resolution approach is necessary. We therefore refrained from characterizing the purified EVs as “exosomes,” as these vesicles emerge from the cell exclusively from an MVB. Moreover, within “exosomes,” subpopulations can be distinguished on the basis of EV marker protein expression (43, 65, 66). These studies show Nef to be present in the tetraspanin CD63- or CD81-positive fraction of EVs. Further investigation of the protein composition of HIV and SIV Nef EVs and how Nef traffics inside the cell is indicated.

The majority of prior studies on EVs and Nef were conducted by using cell culture-based experimental designs, as *in vivo* studies remain limited by the availability of large volumes of body fluids positive for HIV or SIV. It was thus gratifying to be able to detect SIV Nef in routine plasma samples from naturally infected animals. SIV has maintained a zoonotic transmission cycle in macaques for >32,000 years (67). In contrast, HIV emerged in the human population in the late 19th/early 20th century from SIV (68–71). We speculate that the continuous transmission of the virus over tens of thousands of years allowed it ample time to evolve high-affinity interactions with multiple host signaling and vesicle maturation pathways, including those that yield and are mediated by EVs. Characterization of the EV-SIV Nef interaction will complement similar studies of the EV-HIV Nef interaction and add the experimental accessibility of the SIV nonhuman primate model.

This study was also motivated by the controversy surrounding the detection of HIV Nef in EVs. Several groups have reported that HIV Nef is present in EVs (35, 43, 44, 72); others have contested these findings (46). It is worth noting that not all groups isolate EVs in the same manner. We chose to isolate them with the crowding agent polyethylene glycol (PEG) 8000 by a method similar to that described in reference 49, as repeated high-speed ultracentrifugation can disrupt the integrity of small vesicles. Low-speed centrifugation, followed by antibody-mediated affinity purification, retained EV function, as explored by multiple assays. Some have reported that tetraspanin molecules, the targets of our affinity purification, can also be associated with HIV particles, particularly those produced from macrophages (73, 74). We did not detect any Gag carryover after affinity purification, though we cannot rule out the possibility that minute amounts of virus would be present in EVs purified from plasma. Regardless, Gag is not required for Nef incorporation into EVs, as we observed both HIV and SIV Nef proteins in EVs taken from transiently transfected cells. In the tissue culture setting or to evaluate the EV association of individual proteins, single-step purification of EVs by either ultracentrifugation or PEG precipitation is convenient and suitable; however, for *in vivo* samples, a second step is needed to remove coprecipitating particles such as high-density lipoproteins and, in the case of virus infection, virion particles.

Nef was present in EVs from multiple animals at multiple time points after infection, and no correlation with the CD4 count or SIV load was observed until the very end stage of sAIDS in one animal. The heterogeneous population of circulating vesicular bodies *in vivo* can make biochemical characterization difficult. Key differences can include, but are not limited to, surface markers, incorporated proteins and nucleic acids, and different physical characteristics such as size (75–79). Hence, we used TEM and nanoparticle size distribution to ascertain the physical properties of EVs. This study adhered to a recommendation from the International Society for Extracellular Vesicles that defines minimal requirements for defining EVs (80) by using three or more markers to attribute the presence of Nef to EVs in both tissue culture and *in vivo* settings.

The process by which EV-delivered proteins disseminate after delivery is largely unknown. We observed that EVs delivered proteins (labeled green with our pan-EV stain ExoGreen) into punctate structures, which are likely intermediates of the endosomal trafficking network and have been previously observed with Nef localization (81). A well-studied role for Nef in productively infected T cells is the degradation of surface molecules such as CD4 and major histocompatibility complex class I (22, 23, 30, 31). To accomplish this, Nef must localize to the inner leaflet of the plasma membrane. We did not observe Nef dispersion throughout the cytoplasm upon delivery by EVs to CD4-negative, Lck-negative HUVECs, which are the most likely target of EV-transferred Nef. This could be due to the small amounts of Nef being delivered via this mechanism, its diffusion to the point of no longer being detectable by standard fluorescence methods, or the requirement of T lineage-specific cofactors. Identifying the fate of EV-delivered proteins will increase our understanding of how viruses usurp this pathway to deliver virus-encoded factors to neighboring cells and elicit paracrine phenotypes. Overall, this study supports a larger theme whereby viruses utilize EVs to facilitate long-term persistence. The list of viruses that utilize EV/exosome biogenesis or signaling consists of evolutionarily distinct viruses such as herpesviruses, flaviviruses, picornaviruses, and others (5, 11–14, 78, 82). With this study, we propose that lentiviruses such as SIV and HIV also use this pathway of extracellular communication for pathogenesis.

## MATERIALS AND METHODS

### Ethics statement.

All of the macaques used in this study were maintained at the Tulane National Primate Research Center (TNPRC), and care was provided by the staff in accordance with all institutional guidelines and recommendations.

### SIV load assay.

SIV titers and genome copy numbers were determined by qRT-PCR as previously described (83).

### EV isolation.

EVs were isolated from HEK-293 cells, macaque plasma, and PBMC culture supernatant by a method similar to that of Rider et al. (49). In brief, 50 ml of HEK-293 cell supernatant, 3 ml of plasma, or 45 ml of PBMC supernatant was centrifuged at 800 × *g* at 4°C for 10 min to remove cells and cellular debris. The supernatant was then transferred to new tubes and centrifuged at 16,000 × *g* at 4°C for 30 min to pellet larger vesicles such as apoptotic bodies and MVs. Supernatant was collected and filtered through a 0.22-µm filter, and EVs were precipitated out of solution by the addition of PEG 8000 to a final concentration of 40 mg/ml (Fisher Scientific) (stock was made at 400 mg/ml in 1× PBS [pH 7.4] and maintained at 4°C). EVs were allowed to precipitate for >8 h at 4°C during nutation and pelleted at 1,200 × *g* at 4°C for 60 min.

### EV labeling.

To label EVs with the membrane dye DiI, the PEG precipitate was incubated with 1 µM Vybrant CM DiI (Life Technologies, Inc.) and 100 µg/ml RNase A (Roche) in 500 µl of PBS at 4°C for 1 h. As a negative control, 1 µM DiI and 100 µg/ml RNase A were added to a separate tube containing 500 µl of 1× PBS (EV resuspension volume). While the EV solution was incubating with RNase A and Vybrant CM DiI, Sephadex G-75 (GE) columns were equilibrated with 5 volumes of cold 1× PBS. Excess PEG 8000, DiI, RNase A, and non-EV-associated proteins/peptides were removed by adding the EV suspension to equilibrated columns. EVs were eluted from the columns with 1 ml of fresh, cold 1× PBS. EVs were then quantified (see below), and 100-µl aliquots were either added immediately to target cells or placed at −80°C for future use.

For labeling with the membrane-permeating esterase reporter ExoGlow (SBI), the PEG precipitate was incubated with 1× (50 µl) ExoGlow and 100 µg/ml RNase A at 37°C for 10 min. Excess RNase A, nonincorporated dye, and proteins/peptides were removed with Sephadex G-75, and labeled EVs were eluted as outlined above. EVs were quantified and added immediately to target cells, or 100-µl aliquots were placed at −80°C for future use.

For labeling with the self-quenching dye R18 (Thermo Fisher), a similar approach was taken in accordance with Montecalvo et al. (63). In brief, affinity-purified EVs were incubated with 50 µM dye at room temperature for 30 min. Excess dye was removed with G-25 spin columns (GE), and EVs were quantified and then added immediately to target cells (see below).

### EV biophysical characterization.

EVs were diluted in PBS and assayed for concentration (number of particles per milliliter) and size distribution analysis with the ZetaView (ParticleMetrix). Forty to 80 individual particles/frame were observed, and Brownian motion was used to calculate the diameters of individual particles. A total of 11 independent reads from at least three independent samples of each group were used.

### EV affinity capture.

To affinity purify EVs, ExoCap (JSR) beads, which are coated with antibodies directed against CD63 and CD81, were used. One hundred microliters of bead slurry was added to each 100-µl aliquot of EVs. Beads were washed three times with washing/dilution buffer and eluted either with 40 µl of exosome elution buffer or directly with Laemmli protein loading buffer.

### EM.

SIV capsid was purified from the PEG precipitation step through immunoprecipitation with an SIV p27-specific antibody and EVs by CD63/CD81 bead purification. The final product was allowed to adsorb to glow-charged carbon-coated 400-mesh copper grids for 3 min and then stained with 2% (wt/vol) uranyl acetate in water. TEM images were obtained with a Philips CM12 electron microscope at 80 kV and captured on a Gatan Orius camera (2,000 by 2,000 pixels) with Digital Micrograph software (Gatan, Pleasanton, CA).

### Protein analysis.

For total protein analysis, EVs were diluted to equal concentrations and run on polyacrylamide gels. Bands were visualized by silver staining (Thermo Fisher). For immunoblot analysis, EVs and control fractions were loaded onto a polyacrylamide gel and transferred onto a nitrocellulose membrane (GE). Membranes were blocked with 7% dry milk in Tris-buffered saline with Tween 20 (TBST) at room temperature for 1 h. The primary antibodies used were diluted in 7% dry milk in TBST to the following final concentrations: GAPDH (Abcam 9485), 200 ng/ml; flotillin 2 (Cell Signaling 3244), 100 ng/ml; histone H3 (Abcam 1791), 200 ng/ml; SIVmac239 p27 monoclonal (4B2) (AIDS Reagent Database 2321), 1 µg/ml; SIV Nef monoclonal (clone 17.2) (AIDS Reagent Database 2659), 1 µg/ml; CD63 (H-193) (Santa Cruz Biotech sc-15363), 200 ng/ml; CD81 (Q-14) (Santa Cruz Biotech sc-31234), 200 ng/ml; GFP (ab290), 100 ng/ml. Secondary fluorescent antibodies were also diluted in 7% dry milk in TBST to the following final concentrations: donkey anti-rabbit IgG IRDye 800CW (LiCor P/N 926-32213), 100 ng/ml; donkey anti-goat IgG IRDye 680RD (P/N 926-68074), 100 ng/ml; donkey anti-mouse IgG IRDye 680RD (LiCor P/N 926-68072), 100 ng/ml. Peroxidase-labeled secondary antibodies were diluted in TBST to a final concentration of 100 ng/ml. Western blot assay images were obtained with the LiCor Odyssey system or X-ray film.

### Cell culture, transfection, and EV adsorption.

The plasmid encoding HIV Nef-HA was a gift from Warner Green (Addgene plasmid no. 24162). Human CD81-mCherry was a gift from Michael Davidson (Addgene plasmid no. 55012). Plasmids encoding Nef fused to GFP in the pCG vector were a gift from Frank Kirchhoff (84). HEK-293 and human osteosarcoma (U2OS) cells were obtained from the American Type Culture Collection and grown in Dulbecco’s modified Eagle medium (DMEM) supplemented with 10% EV-free fetal bovine serum (Sigma) and 1× Pen/Strep (Gibco) at 37°C at 5% CO_2_.

HEK-293 cells were transfected with 5 μg of plasmid by using Lipofectamine 2000 (Invitrogen). In brief, 5 μg of plasmid was added to 500 μl of unsupplemented DMEM, and 20 μl of Lipofectamine was added to a separate tube containing 500 μl of unsupplemented DMEM. The Lipofectamine mixture was then added to the plasmid mixture, mixed thoroughly, and incubated at room temperature for 15 min prior to dropwise addition to the cells.

To monitor EV adsorption, 2.5 × 10^5^ cells were seeded into six-well tissue culture plates to a total volume of 3 ml in serum-free medium. For cells treated with EVs labeled with Vybrant CM DiI, increasing amounts of labeled EVs (5 × 10^8^ to 5 × 10^10^/ml) were added to the cells and allowed to adsorb for 2 h. Cells were then trypsinized and analyzed by flow cytometry. U2OS cells treated with EVs labeled with ExoGlow Green were grown on coverslips and treated with 10^10^ EVs for 8 h in serum-free medium. Cells were then prepared for fluorescence imaging (see below). For analysis of lipid fusion (R18 fluorescence assay), cells were grown in 96-well tissue culture plates. Twenty-four hours after cell seeding, R18-labeled EVs were added at a concentration of 8 × 10^10^ particles/ml. Fluorescence of cells was monitored with the FLUOstar Optima (BMG Labtech). The mean fluorescence intensity (MFI) of four independent wells was determined. Initial baseline fluorescence readings (no EVs added) were taken 5 min prior to the addition of EVs and arbitrarily set to 1. Fold fluorescence was then calculated for each time point after EV addition. For the wells containing only EVs, a similar approach was taken. At the end of the time course, Triton X-100 was added to a final concentration of 0.1% to burst EVs and release quenched R18.

### Development of stable CD81-mCherry cell lines.

U2OS cells were transfected with CD81-mCherry/Neo vector (Addgene plasmid no. 24162) and selected with 500 μg/ml Geneticin (G418 salt; Gibco). After 7 days of selection, cells were sorted with the FACSAria II at the University of North Carolina Flow Cytometry Core. Highly fluorescent cells were sorted and maintained under continuous selection with medium supplemented with 250 µg/ml Geneticin. The same process was used for the dual CD81-mCherry and CD63-enhanced GFP cell line that was used as a positive control.

### Immunofluorescence assay.

To detect EV-transferred Nef-HA, we performed an immunofluorescence assay. As the primary antibody, we used a mouse anti-HA antibody (ab18181) or normal mouse serum diluted to 1 μg/ml in 5% bovine serum albumin (BSA). For the secondary antibody, we used an Alexa Fluor 647-conjugated goat anti-mouse IgG antibody (ab150115) diluted at 200 ng/ml in 5% BSA. 4',6-Diamidino-2-phenylindole (DAPI) was added to a final concentration of 100 ng/ml, and coverslips were mounted onto slides with Vectashield (Vector Laboratories). Images were taken and processed as indicated below. To detect EV transfer, cells were grown on coverslips, treated with ExoGlow Green EVs (or ExoGlow in PBS filtered through G-75 columns as a negative control), and fixed with 4% paraformaldehyde at room temperature for 15 min. Cells were then washed twice with 1× PBS, permeabilized with 0.5% Triton X-100 at room temperature for 15 min, and again washed twice with 1× PBS. DAPI was added to a final concentration of 100 ng/ml, and coverslips were mounted onto slides with Vectashield (Vector Laboratories).

### Microscopy and three-dimensional reconstruction.

Z-stack images were taken with a Leica DM5500 microscope with a 63× objective and a Retiga-2000RV camera (QImaging). Images were deconvoluted with MetaMorph v 7.8.12.0 (Molecular Devices) and visualized with Imaris v 8.3.1 (Bitplane).

### Flow cytometry.

A total of 5 × 10^5^ U2OS cells grown in a six-well plate were monitored for EV adsorption with the MACSQuant VYB flow cytometry machine. Gates for forward and side scatter were set with FlowJo v10.1, and the percentage of positive cells was calculated.

### Detection of Nef by flow cytometry.

PBMCs were obtained from blood from SIV-naive TNPRC colony animals. CD4^+^ T cells were magnetically isolated from the PBMCs with CD4 microbeads (Miltenyi). These cells were activated with ConA for 2 days and cultured in the presence of 50 U/ml IL-2 (PeproTech). A total of 750,000 CD4^+^ T cells were incubated with 1.5 × 10^10^ EVs (isolated at time points pre- and postinfection with SIVmac239) in a final volume of 250 μl in a 48-well plate. After 4 h, the volume was increased to 1 ml. Cells were harvested 24 h later, washed, fixed, and then permeabilized, and the anti-Nef monoclonal antibody (clone 17.2) and an FITC-labeled anti-mouse secondary antibody were added. Data were collected with a BD LSRII instrument and analyzed with FlowJo v10.0.8. For EV-adsorbed cells, differential gating of EV-positive and EV-negative cells allowed for comparison of Nef expression in the two gates. In some cultures, two ART drugs, AZT and NFV, were added at 100 nM at the beginning of the experiment to prevent virion maturation and *de novo* expression of Nef.

### Infection of PBMCs with SIV from naive macaques.

PBMCs were isolated from 60 ml of blood from each of four SIV-naive macaques. CD8-positive cells were removed by magnetic separation (Miltenyi). CD8-negative cells from the four animals were mixed and cultured with ConA for 2 days prior to infection. ConA was then removed, and the cells were split into four cultures of approximately 90 million each in medium containing 15% exosome-depleted serum and 50 U/ml IL-2. Two of the cultures were maintained with ART drugs; one was maintained with 100 nM AZT, and the other was maintained with 100 nM NFV. Twenty-four hours later, the cells were infected with SIVmac239 at an MOI of 10 and maintained for 5 days. As a control group, one flask was left uninfected. Medium containing the ART drugs was replaced every other day. After 5 days, culture supernatant was removed and clarified for EV characterization as outlined above.
