# Supplementary figures and images for: Nef Secretion into Extracellular Vesicles or Exosomes Is Conserved across Human and Simian Immunodeficiency Viruses
Source: mBio. 2018 Feb 6;9(1):e02344-17. doi: 10.1128/mBio.02344-17 (PMC5801467; doi:10.1128/mBio.02344-17)

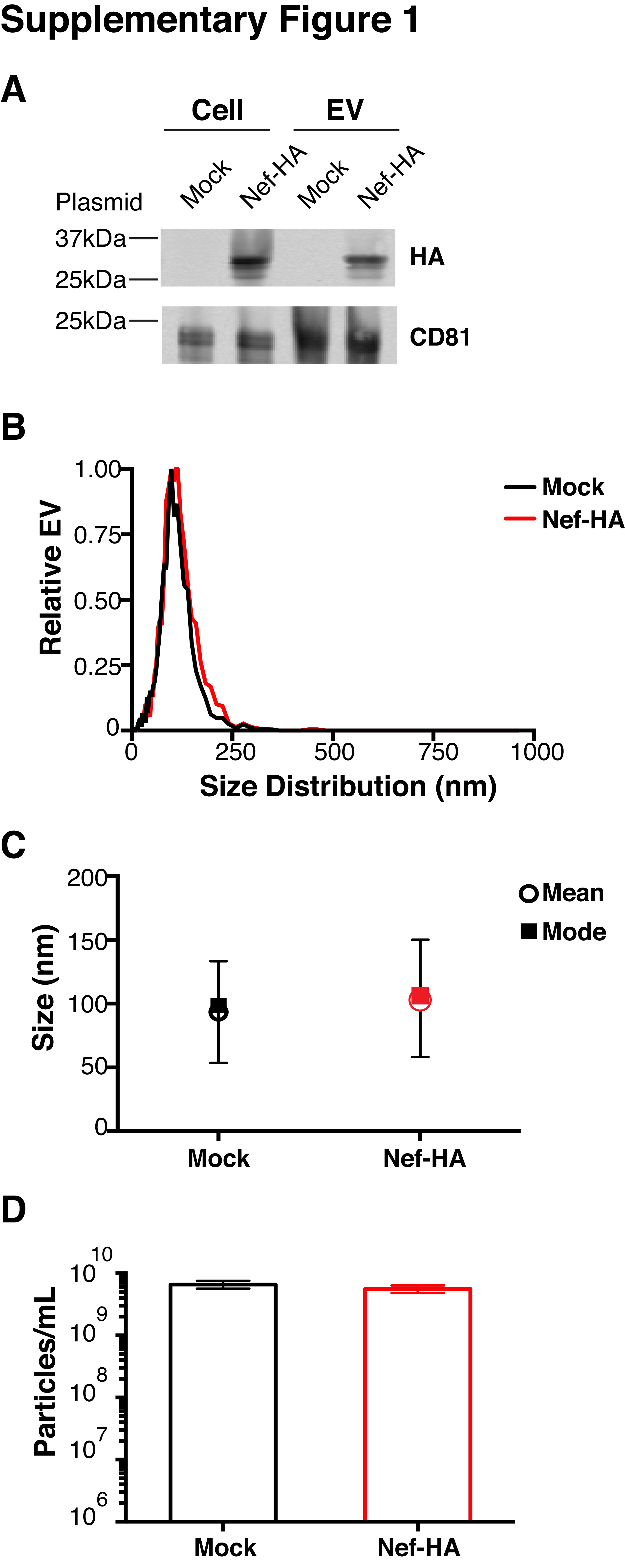

Supplement: FIG S1 [file mbo001183720sf1.tif]

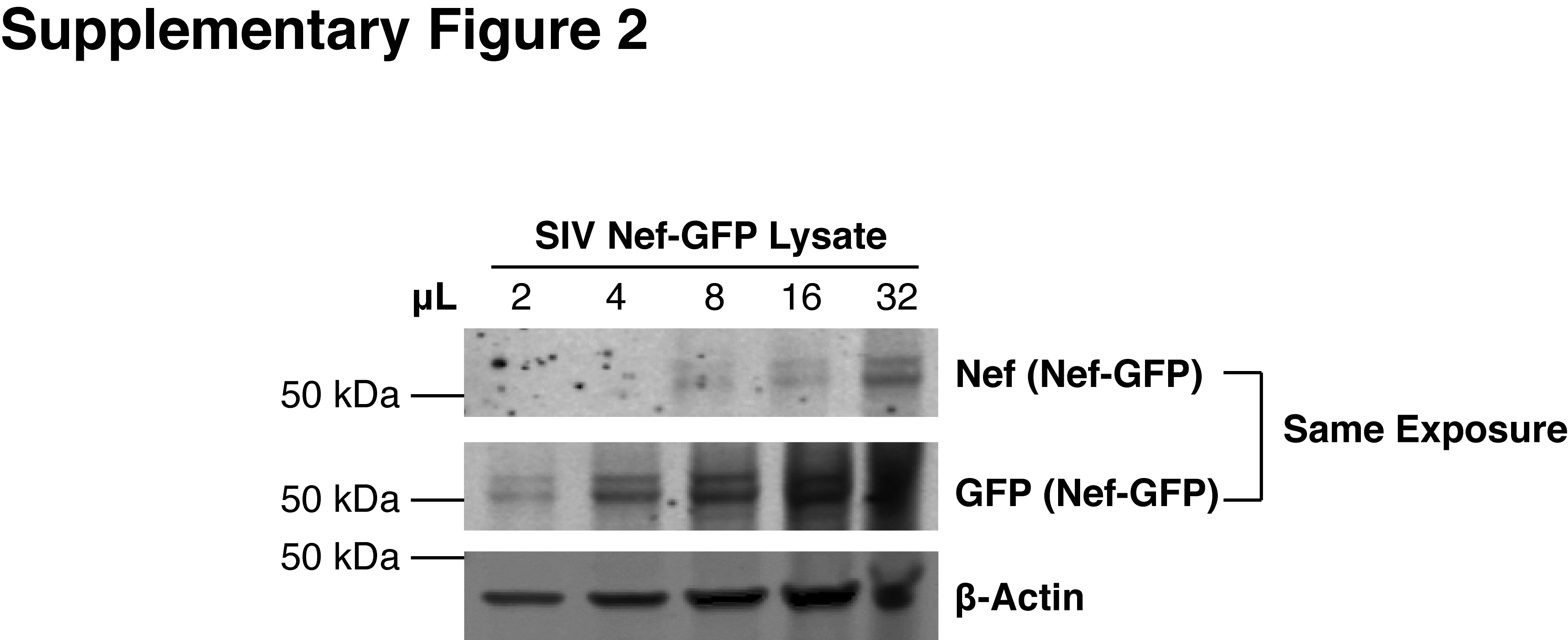

Supplement: FIG S2 [file mbo001183720sf2.tif]
